# Nanoparticles as a Delivery System of Antigens for the Development of an Effective Vaccine against *Toxoplasma gondii*

**DOI:** 10.3390/vaccines11040733

**Published:** 2023-03-25

**Authors:** Carina Brito, Camila Lourenço, Joana Magalhães, Salette Reis, Margarida Borges

**Affiliations:** 1 UCIBIO/REQUIMTE, Laboratory of Biochemistry, Department of Biological Sciences, Faculty of Pharmacy, University of Porto, 4050-313 Porto, Portugal; 2 LAQV, REQUIMTE, Department of Chemical Sciences, Faculty of Pharmacy, University of Porto, 4050-313 Porto, Portugal; 3 Associate Laboratory i4HB—Institute for Health and Bioeconomy, Faculty of Pharmacy, University of Porto, 4050-313 Porto, Portugal

**Keywords:** nanoparticles, adjuvant, immune system, toxoplasmosis, *Toxoplasma gondii*

## Abstract

Nanoparticles include particles ranging in size from nanometers to micrometers, whose physicochemical characteristics are optimized to make them appropriate delivery vehicles for drugs or immunogens important in the fight and/or prevention of infectious diseases. There has been a rise in the use of nanoparticles in preventive vaccine formulations as immunostimulatory adjuvants, and as vehicles for immunogen delivery to target immune cells. *Toxoplasma* is important worldwide, and may cause human toxoplasmosis. In immunocompetent hosts, infection is usually asymptomatic, but in immunocompromised patients it can cause serious neurological and ocular consequences, such as encephalitis and retinochoroiditis. Primary infection during pregnancy may cause abortion or congenital toxoplasmosis. Currently, there is no effective human vaccine against this disease. Evidence has emerged from several experimental studies testing nanovaccines showing them to be promising tools in the prevention of experimental toxoplasmosis. For the present study, a literature review was carried out on articles published over the last 10 years through the PubMed database, pertaining to *in vivo* experimental models of *T. gondii* infection where nanovaccines were tested and protection and immune responses evaluated. This review aims to highlight the way forward in the search for an effective vaccine for toxoplasmosis.

## 1. Introduction

**Figure 1:**
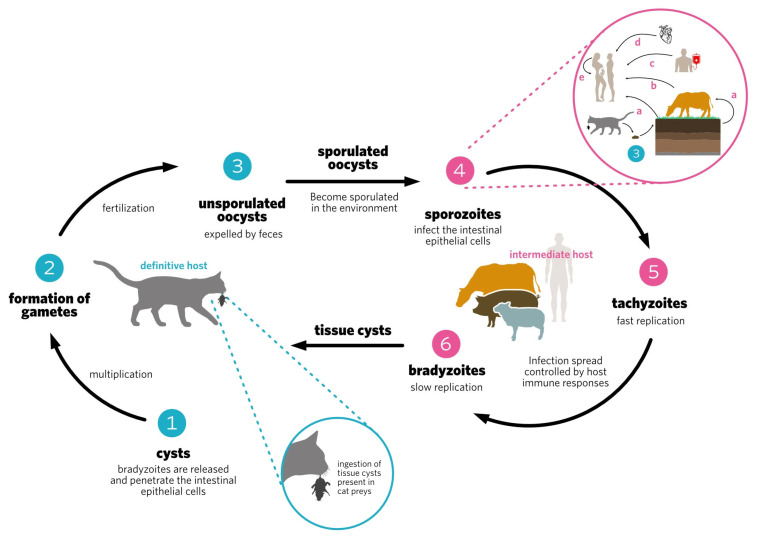
Life cycle of *Toxoplasma gondii*. 1—Consumption of meat containing cysts by the definitive host (Felidae), followed by the release of bradyzoites, and infection of intestinal epithelial cells; 2—After intense multiplication, gametes are formed; 3—After fertilization, unsporulated oocysts are released in the feces of the definitive host; 4—Intermediate hosts are infected by diverse means, such as by the ingestion of sporulated oocysts (**a**) and consequently the sporozoites infect the intestinal cells; 5—Conversion of sporozoites into tachyzoites, the rapidly multiplying form; and 6—Host immune responses contribute to the conversion of tachyzoites into bradyzoites, constituting cysts, a slowly replicating form. There are several pathways of intermediate host transmission: (**a**) ingestion of oocysts present in water, vegetables, or fruits; (**b**) ingestion of tissue cysts present in undercooked meat; (**c**) infection with tachyzoites by blood transfusion; (**d**) infection with cysts through tissue transplantation; and by (**e**) vertical transmission.

*Toxoplasma gondii* (*T. gondii*), has a worldwide distribution [1]. *T. gondii* is an intracellular protozoan parasite of the Apicomplexa phylum, which may cause significant clinical manifestations of toxoplasmosis, especially in immunocompromised individuals, pregnant women, and cattle [2]. It has a complex life cycle (Figure 1) involving sexual reproduction in cats, the definitive host, and asexual reproduction in other warm-blooded animals, the intermediate hosts. There are three infectious development stages: tachyzoites, bradyzoites (in tissue cysts), and sporozoites (within oocysts) [3]. The parasite can be transmitted horizontally or vertically [4]. Parasite reactivation or primary infection during pregnancy can cause congenital toxoplasmosis, leading to severe consequences for the fetus, such as abortion, mental retardation, ocular disease, and hydrocephaly [5]. Immunocompromised patients may also develop severe diseases, such as encephalitis and pneumonitis. Some psychiatric illnesses such as schizophrenia, depression, and bipolar disorder have been associated with *T. gondii* infection [6,7].

Different drugs are used in conventional toxoplasmosis treatment, such as pyrimethamine and sulfadiazine. However, therapeutic adherence of this drug combination is still low since it exhibits severe side-effects and is only active against the tachyzoite form, failing to eliminate the latent forms, such as slow-dividing bradyzoites within tissue cysts [8]. Vaccines are a good alternative to chemical therapeutics. However, it has been difficult to achieve an effective, durable, and safe vaccine against toxoplasmosis. Nowadays, only one vaccine, Toxovax^®^ [9], is licensed for use in sheep and goats. This live attenuated vaccine has some disadvantages including limited shelf life, risk of infection to humans handling the vaccine, and possible virulence reversion [10].

New approaches are needed for human toxoplasmosis prevention. With the rapid development of nanotechnology in biomedicine, nanoparticles (NPs) have become attractive and strong candidates for the prevention of infectious diseases, such as COVID-19, hepatitis B, and toxoplasmosis, among many others [11,12]. In some cases, NPs are immunogenic by themselves, without the need for adjuvants to activate the immune system [13,14]. This literature review aims to provide an update on nanoparticles, the benefits of preventive nanovaccines, to present knowledge on the type of host immune responses developed against *T. gondii* infection, and give an overview of the most recent advances in experimental *in vivo* studies testing nanovaccines against *T. gondii* infection. Overall, this work aims to establish future directions in the search for an effective nanovaccine to prevent toxoplasmosis.

## 2. Revisited Host Immune Responses against *T. gondii*

A balance is established between the immune response developed by the host against the parasite and the evasion strategies triggered by the parasite in such a way that both organisms coexist. Any interference with this balance can lead to a decrease in or even a loss of the functional immune system, such as is the case of *T. gondii* and AIDS co-infected individuals, resulting in uncontrolled parasite replication which may lead to encephalitis and even death [15].

*T. gondii* is usually acquired orally. After ingestion of *T. gondii* tissue cysts or oocysts, bradyzoites or sporozoites, respectively, are released in the small intestine where the intestinal epithelial cells constitute the first line of defense against this parasite [15]. Soon after ingestion and invasion of the intestinal epithelium, chemokines are released by the infected enterocytes which consequently attract neutrophils, dendritic cells (DCs), and monocytes/macrophages to the site of infection [16]. The interaction between these cells plays an essential role in the initiation of the immune responses, leading to the development of adaptive immunity [17].

**Figure 2:**
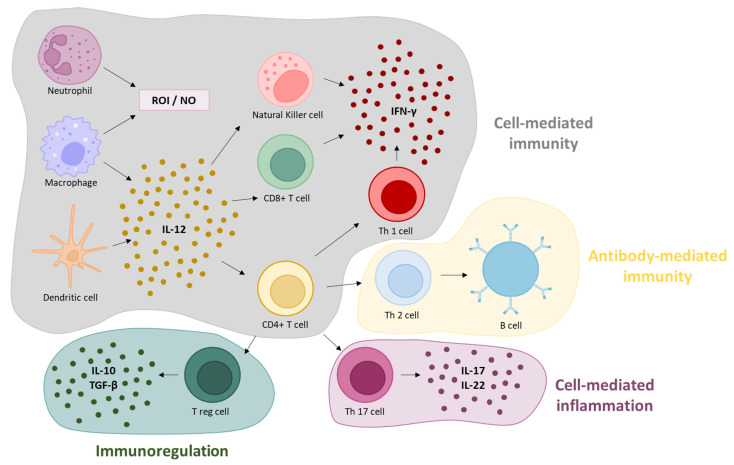
*T. gondii* immune response. Neutrophils and macrophages phagocytize and kill the parasite by releasing antimicrobial compounds and by producing ROI and NO. Macrophages produce IL-12 activating NK cells and T cells to produce INF-γ. DCs phagocyte the parasite and migrate to lymphoid organs where they present their antigens via MHC I or II to CD8^+^ cytotoxic T lymphocytes or CD4^+^ helper T cells, respectively. CD8^+^ CTL will kill the infected cells by apoptosis, and CD4^+^ T cells undergo activation and maturation towards a strong Th1 or Th17 response, through the production of IL-17, IL-21, or IL-22. NK, CD8^+^ T cells and Th1 cells produce IFN-γ, controlling infection. Th2 cells secrete cytokines that stimulate B lymphocytes and consequently the production of antibodies, allowing tachyzoite phagocytosis.

The early infected DCs, macrophages, and neutrophils stimulate the synthesis of IL-12 and induce NKs and T lymphocytes to synthesize INF- γ, which is responsible for the control of infection (Figure 2) [18].

Neutrophils are the first phagocytic cells recruited to the place of infection [17]. They secrete pro-inflammatory chemokines and cytokines that act on the Th1 cell surface receptors, stimulating and attracting other T cells to the infection site [15]. The depletion of neutrophils, during early infection, leads to a Th2 response, since the decreased levels of IFN-γ, IL-12, and tumor necrosis factor- α (TNF-α) lead to a weaker Th1 response, increasing susceptibility [19]. The elimination of the parasites by neutrophils is dependent on IL-17 signaling; the main cytokine responsible for their development and recruitment [20].

DCs are one of the initial antigen-presenting cells at the site of infection, and their maturation and activation are essential in the control of infection. DC’s maturation is characterized by an up-regulation of activation marker expression that leads to naïve T cell activation, proliferation, and differentiation [21,22]. DCs are also able to phagocyte parasites and thereafter migrate to lymphoid organs, where antigen presentation occurs, driving the polarization of the Th response towards Th1, for instance through the production of IL-12 [23]. IL-12 release by DCs is believed to be initiated by the Toll-like receptor’s (TLRs)/IL-1R adaptor protein MyD88. During murine *T. gondii* infection, TLR11 is critical for regulating IL-12 production through TLR11 and MyD88 pathway. It was found that TLR2 and TLR4 are also activated by *T. gondii* with parasite anchors glycosylphosphatidylinositol (GPI) regulating TNF responses and cytokine production, respectively [24].

Macrophages are capable of controlling parasite proliferation through a nitric oxide (NO) dependent mechanism [25]. However, the parasites that manage to resist will penetrate directly into the DC and in this way cross and invade the circulatory and lymphatic system, and disseminate throughout the organism. IFN-γ acts synergistically with TNF-α and increases the production of NO and reactive oxygen species (ROS) in macrophages. IFN-γ is also able to induce iron deprivation essential for parasite multiplication, activate Guanosine triphosphate hydrolase (GTPases), which exposes the parasite to cytosol, and induce tryptophan deprivation preventing parasite growth [18,26].

NKs are also very important in early resistance to *T. gondii* since they recognize and kill infected cells. They are the principal source of IFN-γ early in infection and therefore induce the classical activation of macrophages [27].

CD4^+^ and CD8^+^ T cells contribute to host defense against *T. gondii* through the production of IFN-γ, TNF-α, IL-6, and IL-1. CD8^+^ T cells are effector lymphocytes against the parasite, while CD4^+^ T cells regulate immune responses [15].

CD8^+^ T cells are important mediators involved in the control of chronic infection and prevention of *T. gondii* infection reactivation. This cell population produces IFN-γ, IL-17, and IL-27, associated with the down regulation of the inflammatory response to *T. gondii* [18]. *T. gondii* stimulates the production of CD8^+^ cytotoxic T lymphocytes (CTLs) that can lyse infected cells by apoptosis [28].

To withstand the parasite, a strong T helper 1 cell (Th1)-triggered immune response is required, leading essentially to the production of IFN-γ, IL-12, and IL-23. In contrast, T helper 2 cells (Th2) increase susceptibility to infection involving interleukins such as IL-4, IL-5, and IL-13. The Th2 response is then negatively regulated by IFN-γ and the Th1 response is negatively regulated by IL-4, allowing for a balance in the immune system [26,29]. T helper 17 cells (Th17) are also important because they contribute to the inflammatory response during parasite infection by producing cytokines such as IL-17, IL-21, and IL-22. The cytokine IL-17 is considered one of the main cytokines involved in neutrophil recruitment and is known to regulate neutrophil influx during *T. gondii* infection [30,31]. The cytokine IL-10 can be produced by Th1, Th2, Th17, and regulatory T cells (Treg). IL-10 is an anti-inflammatory cytokine and when produced by Th2 cells can regulate the Th1-triggered immune response associated with downregulation of the pro-inflammatory cytokine IFN-γ. Therefore, by regulating innate and adaptative responses, these cytokines establish homeostasis in the organism, which is essential to controlling *T. gondii* infection [32,33,34]. Transforming growth factor (TGF)-*β,* another key regulatory cytokine, is critical in the regulation of the pathogenic responses that occur during oral infections. TGF-*β* production downregulates the transcription of pro-inflammatory mediators such as IFN-*γ*, iNOS, and TNF-*α* in the intestine as well as modulating the pro-inflammatory responses of lamina propria lymphocytes [35].

The humoral response induced by *T. gondii* results in raised levels of immunoglobulins (IgA, IgM, IgE, and IgG) [36]. Among antibodies, IgM is not only the best activator of the complement system, but also has an excellent ability to agglutinate particulate antigens. Consequently, IgMs have a high level of cytotoxicity against pathogens and can also be used in serological diagnostic techniques. IgG1, IgG2, and IgG3 subclasses are predominantly produced during infection, one of their main roles being to protect the fetus with their ability to cross the placenta in the event of infection during pregnancy [37]. Immunoglobulin A (IgA) is the predominant antibody isotype in the mucosal immune system, which widely exists in the gastrointestinal tract, respiratory tract, vaginal tract, tears, saliva, and colostrum [38]. The IgA comprises two isotypes, IgA1 and IgA2, found predominantly in the serum and digestive tract, respectively [39,40]. There are few studies done regarding IgE, since its appearance is random and associated with the beginning of complications, such as, adenopathy and chorioretinitis due to *T. gondii* reactivations in immune-depressed individuals [41].

These antibodies act on extracellular tachyzoites released by the lysis of infected cells and limit their replication by lysing them in the presence of complement factors, helping opsonization and macrophage phagocytosis [42].

## 3. Nanoparticles Overview

The term NPs refers to particles that, regardless of their constitution, shape, molecular interaction, and therapeutic application, are measured at the nanometer scale (1–100 nm) [43,44]. However, in the scientific literature (especially in biology and medicine), the term is commonly used to describe particles with an average size below 1000 nm [45,46,47]. Indeed, for non-biomedical applications, NPs are considered particles of nanometer size, while in the biomedical sciences they encompass particles from nanometers (10^−9^ m) to micrometers (10^−6^ m) in size, which are biologically active by themselves, or have the ability to target and deliver drugs [48]. One of the areas to benefit most from nanotechnology is medicine. Nanomaterials are useful as contrast agents in imaging and pathology diagnosis, and as a means of modulating cell behavior beneficial to the treatment of several pathologies, such as allergies, cancer, autoimmune diseases, and Alzheimer’s disease [47,49,50]. Examples include drug carriers and preventive or therapeutic vaccines in cancer control [51,52]. NPs therefore enable targeted therapeutics delivery, surpassing biological barriers and causing less damage to healthy cells [52].

### Types of Nanoparticles Used in Vaccination

There are two main categories of NPs used in biomedicine: manufactured (designed for human need and therapeutic applications) and naturally produced (from human and animal tissues, eukaryotic or prokaryotic cells, insects, and plants) [53]. In biomedicine, both types are widely used; however, one of the main differences between natural and manufactured NPs is the fact that the latter exhibit controlled physicochemical properties, since they are purposely designed [53]. Some of the types of nanoparticles used in vaccination are described in Table 1.

Inorganic NPs are almost exclusively derived from unnatural sources, which confers them with characteristics that can be explored and modified according to the intended immunological applications [68]. Some biocompatible inorganic NPs such as gold, carbon, and silica have been explored in vaccine delivery studies with different medical applications, such as targeted drug delivery and enhancing antibody response [55,56,57]. Polymeric-based NPs can have natural or synthetic origin and are currently one of the most investigated types of NPs [54]. A wide range of synthetic polymers are used, such as poly (lactic-co-glycolic acid) (PLGA) and poly (lactic acid) (PLA). Natural polymers such as pullulan, alginate, inulin, and chitosan, have also been used as adjuvants [58]. Liposomes are spherical structures of spontaneous synthesis [59]. These NPs are considered the second most researched vehicle for the delivery of drugs and vaccines in nanomedicine after polymeric NPs [46,54]. Solid lipid NPs (SLN) were developed in the early 1990s as an alternative to traditional liposomes and polymeric NPs [69]. This type of NP includes a lipid matrix composed of lipids such as, triglycerides, phosphoglycerides, fatty acids, hard fats, and waxes, reducing the risk of acute and chronic toxicity [62,63]. Virus-like particles (VLPs) are ideal systems for nano-based vaccines since they show immunogenic properties, thus, avoiding infectious components [46]. Polypropyleneimine (PPI) and polyamido amine (PAMAM) are the most used dendrimers for vaccine delivery [54]. A dendrimer encapsulated with multiple antigens was able to produce a strong antibody and T-cell response against *T. gondii* in mice [70].

## 4. Benefits of Using Nanoparticles in Vaccination

NPs are used as an antigen delivery tool and/or as an immune-stimulant adjuvant to enhance immunity against several pathogens such as *Mycobacterium tuberculosis* and *T. gondii* [54,71].

In traditional vaccines, the formulation is distributed without a specific target in the body. NPs as a vehicle may alter the active substance distribution *in vivo*, since they can be covered with antibodies on their surface, which are capable of recognizing cell-specific receptors, thus, allowing targeted delivery to a desired cell population and preventing potential damage to other cells, and consequently to other tissues [72]. Additionally, these NPs have a depot effect, when administered by intramuscular route, keeping the antigen in the tissue area adjacent to the administration site long enough to exert the necessary function. This allows a gradual release of the antigen, thereby increasing the exposure time to the immunogen by antigen-presenting cells (APCs). Therefore, APCs will increase their ability to present the antigen and induce an efficient T-cell response [73,74].

Adjuvants are chemical or biological compounds that stimulate the immune system against the administered antigen, thus, increasing the effectiveness of the vaccine [75]. Among the most common adjuvants, aluminum (Alum)-based compounds, such as amorphous Alum hydroxy-phosphate sulphate (AAHS), Alum hydroxide, Alum phosphate, and potassium Alum sulphate, are the most used in conventional human vaccines [76]. Synthetic oligodeoxynucleotides (ODNs) are also adjuvants; they contain unmethylated CpG motifs and can trigger cells expressing TLR9 inducing a Th1 response and proinflammatory cytokines. Overall, ODNs improve APC function and boost the humoral and cellular vaccine-specific immune responses [77]. However, these adjuvants have several disadvantages, such as the need to be stored at low temperatures or the possibility of allergic reactions at the injection site [78]. NPs are an alternative to the use of such adjuvants, with equal or high immune system-stimulating ability [75,79].

Since NPs share structural and size characteristics with viruses and bacteria they can mimic the process of a natural infection increasing the uptake of antigens by APCs, and consequently immune response initiation [72]. Studies have shown that macrophages and DCs are capable of capturing cationic NPs since their positive charge is attracted by the negative charge of the membrane surface of these cells [80]. The NPs can also be conjugated with antibodies specific to cell receptors, as previously mentioned, enabling NP internalization, as was shown for Herceptin-coated gold NPs endocytosed after interaction with the membrane HER2 receptor, used in breast cancer [81].

NPs are also used to improve the solubility of hydrophobic compounds, thus, obtaining a solution for parenteral administration, preventing antigen degradation and allowing the stabilization of a wide range of therapeutic agents such as proteins, peptides, and nucleic acids, which leads to a reduction in doses of effective vaccines [75].

## 5. Recent Advances in the Use of Nanoparticles for *T. gondii* Vaccination

A search using the terms “Nanoparticles”, “Vaccine”, and “*Toxoplasma gondii*” and the filter “last 10 years” enabled 40 articles to be identified. Among these, 16 corresponded to reviews concerning experimental NPs tested for the diagnosis and treatment of toxoplasmosis. We found 24 articles detailing studies focused on the development of nanovaccines against *T. gondii* infection.

Several studies have been carried out into the development of a vaccine against *T. gondii* (Table 2). However, an effective vaccine able to confer effective immunity against latent infection (elimination of tissue cysts) remains a challenge [82]. Recently, new approaches have been made in vaccination strategies, such as the use of NPs, which have been assessed mostly in rodents, showing promising results [83]. An ideal vaccine to control toxoplasmosis and prevent the development of chronic tissue cysts should induce a Th1- type immune response, since it has been shown that the INF-γ-secreting CD8^+^ T lymphocyte is the main immune cell population involved in the long-term protective immunity against this disease [82]. Mucosal immunization routes, such as intranasal and/or intraoral, have been shown to induce effective protection when compared to systemic immunization routes, such as intramuscular or intravenous [84].

NP development has emerged as a novel vaccine platform, currently constituting a strategy to protect antigens from proteolytic degradation, ensuring a successful uptake by cells and inducing an effective immune response [83]. Different NP antigen delivery strategies have been studied, such as DNA and ribonucleic acid (RNA) vaccines, as well as protein and recombinant subunit vaccines [83]. Nowadays, DNA and RNA vaccines have been shown to be the most efficient platforms, able to induce *anti-T. gondii* immune responses, and easily produced at a low cost [85]. Indeed, calcium phosphate NPs (CaPNs) encapsulated with DNA or RNA coding for dense granule protein 14 (GRA14) have been shown to increase *T. gondii* specific IgG1 and IgG2a antibody responses and lymphocyte proliferation [86]. Similar NPs coding dense granule protein 7 (GRA7) also showed a strong cellular immune response, with a higher IgG2a-to-IgG1 ratio and higher IL-12 and INF-γ production [87]. Immunization using the mice model, with a modified dendrimer vaccine with mRNA replicons encoding dense granule protein 6 (GRA6), rhoptry protein 2A (ROP2A), rhoptry protein 18 (ROP18), surface antigen 1 (SAG1), surface antigen 2 (SAG2) and apical membrane antigen 1 (AMA1), led to protection against lethal infection [70]. It has been shown that a significant percentage of mice immunized with lipid nanoparticles encapsulated with nucleoside-triphosphatase II (NTPase II) survived post-challenge with *T. gondii* parasites [88]. Other similar lipid nanoparticles encapsulated with a plasmid encoding GRA15 led to a significantly higher production of specific IgG1 and IG2c antibodies and consequently higher survival rate compared to the controls [89]. Cocktail DNA vaccines of pcROM4 + pcGRA14 coated with CaPNs boosted immune responses and increased the protective efficacy against acute toxoplasmosis compared to cocktail DNA vaccine without CaPNs [90].

Protein and recombinant subunit vaccines are also extremely safe with low side effects since proteins are highly purified. A wide array of antigens has been tested, ranging from antigenic epitopes to total *T. gondii* antigenic extract (TE) [83]. Mice immunized with porous NPs containing TE have been shown to induce a Th1/Th17 immune response able to prolong mouse survival and drastically reduce brain cyst counts [91]. Polymeric NPs loaded with *T. gondii* histone H2A1 conferred mice with protection against infection, prolonging their survival and the production of Th1 cytokines [92]. A similar study using PLGA NPs containing T and B cell epitopes of AMA1, GRA4, ROP2, and SAG1, adjuvanted with potassium Alum sulphate, induced a stronger Th1 immune response in mice, compared to immunization solely with antigens [93]. Another study showed that intranasal immunization of mice with maltodextrin-based NPs (DGNPs) containing TE conferred significant protection against chronic and congenital toxoplasmosis [94]. This vaccine was later shown to induce protection against latent toxoplasmosis and transplacental transmission using the sheep model [95]. Some immunization studies with different types of nanoparticles (CaPNs, PLGA, and Chitosan) encapsulated with a variety of *T. gondii* recombinant proteins, namely MIC3, ROP8, and SAG1, showed similar results, with higher levels of specific IgA and IgG2a, leading to a Th1 response and consequently increased survival rate [96,97,98,99,100]. In addition to the use of parasite membrane proteins, the excretory secretory antigens (ESA) of *T.gondii* have also been used for vaccine development, since they have proved to play important roles in the immune escape and pathogenesis of the parasite, and the results showed increased levels of IFN-γ and IgG, as well as a reduction in the parasite load [101,102,103].

Studies concerning *T. gondii* describing the use of VLPs are limited. Nevertheless, some VLP-based vaccines are already commercially available for several human viral diseases, such as Epaxal^®^ for hepatitis A virus, Gardasil^®^ for human papillomavirus, and GenHevac B^®^ for hepatitis B virus, among others [104]. The advantages of VLPs described include: safety; small size, allowing rapid traffic into the lymph nodes and consequent induction of a prompt immune response; and their repetitive antigen presentation, promoting a powerful immune response [105]. It has been shown that immunization with VLPs containing *T. gondii* inner membrane complex subcompartment protein 3 (IMC ISP3) with influenza matrix protein 1 (M1) as a core protein conferred mice with protection against *T. gondii* ME49 infection [106]. Thereafter, multiple studies have shown the efficacy of VLP vaccines containing different antigens in enabling protection against the ME49 strain. Finally, VLPs expressing ROP4 or ROP13 conferred complete survival against the challenge with the ME49 strain and reduced cyst numbers [107]. It must be highlighted that these VLPs were developed containing the self-assembling viral protein M1 determinant for VLP generation in which M1 is the main force for viral budding and particle formation. It is assumed that *T. gondii* proteins will be on the surface of VLPs [106,107].

It is important to mention that almost all studies of nanovaccines in toxoplasmosis used the mouse as a study model. Therefore, future pre-clinical trials should be extended to other animal models. There should also be a harmonization of the immune parameters assessed in these studies in order to support the studies carried out.

**Table 2:** Recent studies carried out using NPs for the development of vaccines against *T. gondii* infection.

Type of NPs	Antigen	Doses	Administration Route	Animal/Cell Model	Outcome	Reference
Dendrimers	mRNA replicons encoding GRA6, ROP2A, ROP18, SAG1, SAG2A and AMA1 proteins	1	IM	C57BL/6 and BALB/c mice	Induction of CD8^+^ T cell responses	[70]
CaPNs or Alum	pcGRA14 and rGRA14	3	SB and IM	BALB\c mice	High levels of IgG, IgG2a and IFN-γ for CaPNs immunization and high levels of IgG1 and IL-4 for Alum hydroxide immunization	[108]
PLGA or Chit	rTgH2A1	1	SB	Institute of cancer research (ICR) mice	High levels of IgG2a	[92]
PLGA	Peptide sequence of SAG1	-	*-*	Gastric adenocarcinoma (AGS) cell line	Molecular docking of peptide and MHC molecules	[109]
DGNP	TE	3	IN	CBA/J mice	Induction of Th1/Th17 immune responses. Higher levels of total IgG detected	[91]
PLGA and Alum	Recombinant fusion protein with epitopes of SAG1, AMA1, ROP2 and GRA4	3	IP	BALB\c mice	Higher levels of IgG2a for PLGA NPs and higher levels of IgG1 for Alum NPs	[93]
CaPNs	pcROM4 + pcGRA14	3	IM	BALB\c mice	Higher levels of INF-γ and IgG2a and prevalence of Th1 immune response	[90]
DGNP	TE	2	IN and ID	Sheep breeds Préalpes du Sud	Specific Th1 cellular immune response (IFN-γ and IL-12); IL-10 regulated the Th1 response for ID but not IN vaccination; IN vaccination conferred protection against latent toxoplasmosis	[95]
CaPNs	Plasmid encoding GRA14	3	IM	BALB\c mice	Increased levels of IgG1 and IgG2a. High levels of IFN-γ; Decrease of parasite load in mice tissues	[86]
CaPNs	Multi epitope MIC3, ROP8 and SAG1	3	SB	BALB\c mice	Survival increase	[96]
VLPs	IMC proteins	2	IN	BALB\c mice	Reduced cyst load and size in the brain. IgA and IgG detection in feces and intestines; Mixed Th1/Th2 cytokines and CD4^+^/CD8^+^ T cells	[106]
PLGA	rROP18	3	IN	Swiss Webster mice	Elevated responses of specific IgA and IgG2a	[97]
Lipid NPs	RNA vaccine encoding *T.gondii* NTPase-II protein	2	IM	BALB\c and ICR mice	High IgG antibody titters and IFN-γ production	[88]
PLGA	rSAG1	3	IN	Swiss Webster mice	Elevated responses of IgA and IgG2a	[98]
PLGA	rSAG1 and TLR ligands	2	SB	CBA/J mice	Potentiated Th1 humoral response; Brain cyst reduction	[99]
Alginate	*T.gondii* ESA	3	IP	Swiss albino mice	Reduction in mice mortality; Increased levels of INF-γ and IgG antibody	[101]
Lipid NPs	Plasmid encoding GRA 15	3	SB	C57BL/6J	Higher survival rate; Higher production of specific IgG1 and IG2c antibodies	[89]
CaPNs	Plasmid encoding GRA 7	3	-	BALB\c mice	Elevated levels of IgG; Higher IgG2a-to-IgG1 ratio; Elevated IL-12 and IFN-γ production and low IL-4 levels; Higher level of splenocyte proliferation and a significantly prolonged survival time and decreased parasite burden	[87]
Chit	rSAG1	3	SB	BALB\c mice	Th1/Th17 biased cellular and humoral immune responses; Increased production of IFN-γ, IL-17, IL-12, IL-4, IFN-γ/IL-4 ratio, IgG, IgG2a; Increased survival time.	[100]
PLGA and Chit	Plasmid encoding TgSDRO	2	IM	BALB\c mice	Induced Th1/Th2 cellular and humoral immunity; Promoted the maturation and MHC expression of dendritic cells, and enhanced the percentages of CD4^+^ and CD8^+^ T lymphocytes	[110]
SAPNs	*ToxAll*	3	IM	HLA mice	High IFN-γ secretion; Reduced parasite numbers in the brains; Enhanced survival	[111]
Mannose-modifed nanoliposome	ESA	2	-	BALB\c mice	Increased IL-12 expression; Increased survival rates	[102]
Liposome	ESA	3	IP	BALB\c mice	Increased IgG antibody; Parasite load reduction in the blood and brain tissue	[103]
DGNPs	TE	2	IN	CBA/J mice	Placental cellular Th1 response; Pups born to vaccinated infected dams had significantly fewer brain cysts, no intraocular inflammation and normal growth	[94]

CaPNs: calcium phosphate nano-adjuvant; Alum: aluminum hydroxide nano-adjuvant; Chit: chitosan; DGNP: porous maltodextrin-based with lipid core nanoparticles; pcGRA14: GRA14 plasmid clones; rGRA14: GRA14 recombinant protein; rTgH2A1: recombinant *T. gondii* H2A1 histone; TE: total extract of *T. gondii* antigens; pcROM4: ROM4 plasmid clones; IMC: inner membrane complex; ESA: Excretory–secretory antigens; rROP18: ROP18 recombinant protein; NTPase-II: nucleoside thiophosphate hydrolase II; rSAG1: SAG1 recombinant protein; TgSDRO: T. gondii oxidoreductase from short-chain dehydrogenase/reductase family; *ToxAll:* five CD8^+^ T cell eliciting HLA-A*11:01 binding protective epitopes, one CD8^+^ HLA-B*07:02, four CD8^+^ HLA-A*02:01, a pan-allelic CD4 epitope, and a MIC1 B cell epitope; SAPNs: self-assembling protein nanoparticles; IM: intramuscular; SB: subcutaneous; IN: intranasal; IP: intraperitoneal; ID: intradermal.

## 6. Concluding Remarks

Development of a vaccine against toxoplasmosis can be challenging, as is proved by the fact that a vaccine for clinical use is still unavailable. The use of multi-antigenic vaccines appears to be more efficient compared to single antigen ones [83]. Therefore, the use of highly immunogenic antigens combined with NPs that might be used as adjuvants and delivery systems appears promising for the future development of an effective vaccine. In addition, the immunogenicity of these nano-formulations can be improved by conjugating them with immunostimulatory molecules, such as mannose or CpG oligonucleotides, which can facilitate their recognition and uptake by APCs. NPs can also be designed for non-invasive administration, such as intranasal immunization, the efficacy of which has been proved in several studies [112,113,114]. Furthermore, NPs provide a prolonged delivery of vaccine antigens, and the possibility of a single-dose vaccine. Currently, the development of nanovaccines aims not only to target immune cells to prevent disease but is also able to exert an effective therapeutic activity in already established diseases.

In recent years, a wide variety of NPs have been used in the production of vaccines against toxoplasmosis. Polymeric NPs have been investigated most in this field since they are easy to prepare, biocompatible, very stable, have low cytotoxicity and allow surface properties to be adjusted as necessary. Within the different polymers used, PLGA is the most common, and several *in vitro* and *in vivo* studies prove its strong immune-stimulatory property. Therefore, the proven advantages of polymeric NPs make them good candidates for further preventive and therapeutic applications.

## Figures and Tables

**Table 1:** Different types of nanoparticles used in vaccination.

Type of NPs	Constitution	Advantages	References
Inorganic	Gold, carbon, silica, calcium phosphate (CaP), among others.	Rigid structure, controlled synthesis, low production cost, reproducibility, and safety.	[54,55,56,57]
Polymeric	Synthetic polymers like poly (lactic-co-glycolic acid) (PLGA) and poly (lactic acid) (PLA); and natural polymers like pullulan, alginate, inulin, and chitosan.	Easy to produce, biodegradable, biocompatible, exhibit low cytotoxicity and their surface properties are easily adjusted as required.	[54,58]
Liposomes	Spherical structures of spontaneous synthesis, formed by biodegradable and non-toxic phospholipids and cholesterol around an aqueous nucleus.	Transport of hydrophobic molecules wrapped in the phospholipid bilayer, or hydrophilic molecules incorporated in the aqueous core. Liposomes can also be modified, when they are conjugated with target ligands or by adding cholesterol on their surface, optimizing their ability to bind to a target cell and their entry into it. These NPs can also incorporate glycoproteins of a viral envelope, creating virosomes.	[54,59,60,61]
Solid lipid nanoparticles (SLN)	Solid lipid dispersed in an aqueous solution that contains a stabilizer. The lipid matrix composed of lipids such as triglycerides, phosphoglycerides, fatty acids, hard fats, and waxes.	Controlled drug release and targeted drug release with increased stability.	[62,63]
Virus like particles (VLPs)	Assembled viral proteins that do not contain genetic viral material. Despite that, they still have an antigenic character like parental viruses, as they mimic them in shape and structure. Concerning VLP synthesis, first the viral structural genes are cloned. Then, viral structural self-assembling proteins are expressed in prokaryotic (bacteria or yeast) or eukaryotic (baculovirus, plant and mammalian cells) expression systems. Finally, to obtain purified VLPs, purification steps like chromatography or ultracentrifugation are needed.	Ideal systems for nano-based vaccines since they display immunogenic properties, avoiding infectious components.	[46,64,65]
Dendrimers	Three-dimensional, mono-dispersed, highly branched, star-shaped macromolecules with nanometre scale dimension, made up of a mixture of amines and amides. Their molecular architecture consists of three different domains: a central core; branches; and terminal functional groups, in the outer surface.	High loading capacity, high bioavailability of the attached drug and high penetrability of biological barriers and cell membranes.	[54,66,67]

## Data Availability

Data sharing not applicable.
